# Supplementary material for: Effect of Promoter Architecture on the Cell-to-Cell Variability in Gene Expression
Source: PLoS Comput Biol. 2011 Mar 3;7(3):e1001100. doi: 10.1371/journal.pcbi.1001100 (PMC3048382; doi:10.1371/journal.pcbi.1001100)
Supplement: Text S1 — Mathematical derivations and supplementary information. A derivation of all equations in the text is presented, together with its corresponding tables and figures. (2.04 MB DOC) [file pcbi.1001100.s001.doc]

**SUPPLEMENTARY INFORMATION**

**EFFECT OF PROMOTER ARCHITECTURE ON THE CELL-TO-CELL VARIABILITY IN GENE EXPRESSION**

**Alvaro Sanchez^1^, Hernan G. Garcia^2^, Daniel Jones^2^, Rob Phillips^3,4^, Jane Kondev^5^**

^1^Graduate Program in Biophysics and Structural Biology and ^5^Department of Physics, Brandeis University, Waltham, MA.

^2^Department of Physics, ^3^Department of Bioengineering and ^4^Department of Applied Physics, California Institute of Technology, Pasadena, CA.

**The moments of the mRNA probability distribution**

We start by considering the same mechanism as in the text (see figure 1), in which the promoter switches between one active and one inactive state. There are only two stochastic variables in the model: the number of mRNA transcripts per cell (*m*), and the state of the promoter which reflects which transcription factors are bound where. The promoter state is always a discrete and finite stochastic variable (*s*) (for an example, see figure 1a). The example in figure 1a illustrates the simplest model of transcriptional activation by a transcription factor.

When the activator is bound to the promoter (state 1) mRNA is synthesized at rate $r_{1}$. When the activator is not bound (state 2) mRNA is synthesized at a lower rate $r_{2}$. The promoter switches stochastically from state 1 to state 2 with rate $k_{A}^{off}$, and from state 2 to state 1 with rate $k_{A}^{on}$. Each mRNA molecule is degraded with rate $\gamma$.

The time evolution for the joint probability of having the promoter in states 1 or 2, with mRNAs in the cell (which we write as $p(1,m)$ and $p(2,m)$, respectively), is given by a master equation, which we can build by listing all possible reactions that lead to a change in cellular state, either by changing or by changing (figure 1b). The master equation takes the form:

$$\begin{matrix} \frac{d}{dt}p(1,m)=-k_{A}^{off}p(1,m)+k_{A}^{on}p(2,m)-r_{1}p(1,m)-\gamma mp(1,m)+r_{1}p(1,m-1)+\gamma(m+1)p(1,m+1), \\ \frac{d}{dt}p(2,m)=k_{A}^{off}p(1,m)-k_{A}^{on}p(2,m)-r_{2}p(2,m)-\gamma mp(2,m)+r_{2}p(2,m-1)+\gamma(m+1)p(2,m+1)\text{ .} \end{matrix}$$

Inspecting this system of equations, we notice that by defining the vector:

$$\vec{p}(m)=\left(\begin{matrix} p(1,m) \\ p(2,m) \end{matrix}\right),$$

and the matrices

$$\hat{K}=\left[\begin{matrix} -k_{A}^{off} & k_{A}^{on} \\ k_{A}^{off} & -k_{A}^{on} \end{matrix}\right]\text{ ; }\hat{R}=\left[\begin{matrix} r_{1} & 0 \\ 0 & r_{2} \end{matrix}\right]\text{ ; }\hat{I}=\left[\begin{matrix} 1 & 0 \\ 0 & 1 \end{matrix}\right],$$

we can rewrite the system of equations (1) in matrix form.

$$\frac{d}{dt}\vec{p}(m)=\left[\hat{K}-\hat{R}-m\gamma\hat{I}\right]\vec{p}(m)+\hat{R}\text{ }\vec{p}(m-1)+(m+1)\gamma\hat{I}\text{ }\vec{p}(m+1)\text{ .}$$

This approach can be generalized to any mechanism of transcriptional regulation at the promoter level. The only difference between the mechanisms rests on the particular dimensionality and form of the three matrices defined above. Examples of those matrices for all of the architectures and mechanisms investigated on this paper are given in Table S1 in Text S1. In steady state, the left hand side of equation (4) is equal to 0:

$$0=\left[\hat{K}-\hat{R}-m\gamma\hat{I}\right]\vec{p}(m)+\hat{R}\text{ }\vec{p}(m-1)+(m+1)\gamma\hat{I}\text{ }\vec{p}(m+1)\text{ .}$$

In order to find the first two moments of the steady state mRNA probability distribution, we follow the same strategy as in references [1,2]: we multiply both sides of equation (5) by $m$ and $m^{2}$respectively, and then sum over all values of $m$, from 0 to$\infty$. We start from the first moment of the mRNA distribution, which requires us to multiply equation (5) by and then sum: $\begin{matrix} \sum_{m=0}^{\infty} m\left(\left[\hat{K}-\hat{R}-m\gamma\hat{I}\right]\vec{p}(m)+\hat{R}\text{ }\vec{p}(m-1)+(m+1)\gamma\hat{I}\text{ }\vec{p}(m+1)\right)=\sum_{m=0}^{\infty} m\text{ }\hat{K}\text{ }\vec{p}(m)-\sum_{m=0}^{\infty} m^{2}\gamma\hat{I}\text{ }\vec{p}(m) \\ -\sum_{m=0}^{\infty} m\text{ }\hat{R}\text{ }\vec{p}(m)+\sum_{m=0}^{\infty} m\text{ }\hat{R}\text{ }\vec{p}(m-1)+\sum_{m=0}^{\infty} m\text{ }(m+1)\text{ }\gamma\hat{I}\text{ }\vec{p}(m+1)\text{ .} \end{matrix}$

Since none of the three matrices$\hat{K}$, $\hat{R}$and $\hat{I}$are functions of *m*, they can be taken out of the sums, and we find:

$$0=\hat{K}\sum_{m=0}^{\infty} m\text{ }\vec{p}(m)-\gamma\hat{I}\sum_{m=0}^{\infty} m^{2}\vec{p}(m)-\hat{R}\sum_{m=0}^{\infty} m\text{ }\vec{p}(m)+\hat{R}\sum_{m=0}^{\infty} m\text{ }\vec{p}(m-1)+\gamma\hat{I}\sum_{m=0}^{\infty} m\text{ }(m+1)\text{ }\vec{p}(m+1)\text{ .}$$

It will be convenient in what follows to define the following vectors of partial moments of the mRNA probability distribution:

$$\begin{matrix} \vec{m}_{(0)}=\sum_{m=0}^{\infty} m^{0}\vec{p}(m)=\left(\begin{matrix} \sum_{m=0}^{\infty} m^{0}p(1,m) \\ \sum_{m=0}^{\infty} m^{0}p(2,m) \end{matrix}\right)=\left(\begin{matrix} p(1) \\ p(2) \end{matrix}\right), \\ \vec{m}_{(1)}=\sum_{m=0}^{\infty} m\text{ }\vec{p}(m)=\left(\begin{matrix} \sum_{m=0}^{\infty} m\text{ }p(1,m) \\ \sum_{m=0}^{\infty} m\text{ }p(2,m) \end{matrix}\right), \\ \vec{m}_{(2)}=\sum_{m=0}^{\infty} m^{2}\text{ }\vec{p}(m)=\left(\begin{matrix} \sum_{m=0}^{\infty} m^{2}\text{ }p(1,m) \\ \sum_{m=0}^{\infty} m^{2}\text{ }p(2,m) \end{matrix}\right)\text{ .} \end{matrix}$$

The usefulness of these vectors of partial moments of the mRNA distribution lies in the fact that they are related to the moments of the probability distribution. For instance, the mean mRNA is given by

$$\left\langle m\right\rangle=\sum_{s=1}^{2} \sum_{m=0}^{\infty} m\text{ }p(s,m)=\sum_{m=0}^{\infty} m\text{ }p(1,m)+\sum_{m=0}^{\infty} m\text{ }p(2,m)\text{ .}$$

If we define, again for convenience, the vector $\vec{u}=\left(1,1\right)$, we find that the mean of the mRNA distribution is related to the vectors of partial moments by $\left\langle m\right\rangle=\vec{u}\text{ }\vec{m}_{(1)}$. Following this example, it is also straightforward to prove that the second moment of the mRNA distribution is given by: $\left\langle m^{2}\right\rangle=\vec{u}\text{ }\vec{m}_{(2)}$.

Given these definitions, we return to equation (7) which we can now write as:

$$\hat{K}\text{ }\vec{m}_{(1)}-\gamma\hat{I}\text{ }\vec{m}_{(2)}-\hat{R}\text{ }\vec{m}_{(1)}+\hat{R}\sum_{m=0}^{\infty} m\text{ }\vec{p}(m-1)+\gamma\hat{I}\sum_{m=0}^{\infty} m\text{ }(m+1)\text{ }\vec{p}(m+1)=0\text{ .}$$

We can re-arrange terms in the last two sums so that we write them as operations on the vectors of partial moments of the probability distributions. For instance, by making the change of variables: $m\to m+1$ , and taking into account the fact that the number of mRNA molecules inside the cell can never fall below 0 (so that $\vec{p}(-1)=0$), we find:

$$\sum_{m=0}^{\infty} m\text{ }\vec{p}(m-1)=\sum_{m=0}^{\infty} (m+1)\text{ }\vec{p}(m)=\vec{m}_{(1)}+\vec{m}_{(0)}\text{ .}$$

Similarly, by making the change of variables $m+1\to m$, the last sum takes the simpler form:

$$\sum_{m=0}^{\infty} m(m+1)\text{ }\vec{p}(m+1)=\sum_{m=0}^{\infty} m(m-1)\text{ }\vec{p}(m)=\vec{m}_{(2)}-\vec{m}_{(1)}\text{ .}$$

Entering these results into equation (10), we finally find:

$$\hat{K}\text{ }\vec{m}_{(1)}-\gamma\hat{I}\text{ }\vec{m}_{(2)}-\hat{R}\text{ }\vec{m}_{(1)}+\hat{R}\left(\vec{m}_{(1)}+\vec{m}_{(0)}\right)+\gamma\hat{I}\left(\vec{m}_{(2)}-\vec{m}_{(1)}\right)=\hat{K}\text{ }\vec{m}_{(1)}-\gamma\hat{I}\text{ }\vec{m}_{(1)}+\hat{R}\text{ }\vec{m}_{(0)}\text{ .}$$

The vector of partial moments is therefore the solution to the matrix equation:

$$\left(\hat{K}-\gamma\hat{I}\right)\text{ }\vec{m}_{(1)}+\hat{R}\text{ }\vec{m}_{(0)}=0\text{ .}$$

The final step is to multiply both sides of equation (14) by the vector $\vec{u}=(1,1)$. Because of how it was constructed (i.e. *p(1,m)*s loss is *p(2,m)*s gain during transitions between promoter states), the matrix$\hat{K}$ has the property that the sum of the elements of any one of its columns is always 0. Therefore, we find that $\vec{u}\text{ }\hat{K}=0$. The matrix is diagonal, so if we multiply matrix $\hat{R}$ on the left by vector , we get a vector that is equal to the list of diagonal elements of matrix . Thus, we define the vector $\vec{r}=\left(\hat{R}_{11},\hat{R}_{22}\right)=\left(r_{1},r_{2}\right)$, as the vector for which it is true that $\vec{u}\text{ }\hat{R}=\vec{r}$. Finally, the identity matrix is $\hat{I}=\left[\begin{matrix} 1 & 0 \\ 0 & 1 \end{matrix}\right]$ . Therefore, multiplying on the left by the vector leads us to: $\vec{u}\text{ }\hat{I}=\vec{u}$. Therefore, when we multiply equation (14) by the vector we find:

$$0=\vec{u}\text{ }\hat{K}\text{ }\vec{m}_{(1)}-\vec{u}\text{ }\gamma\hat{I}\text{ }\vec{m}_{(1)}+\vec{u}\text{ }\hat{R}\text{ }\vec{m}_{(0)}=-\gamma\vec{u}\text{ }\vec{m}_{(1)}+\vec{r}\text{ }\vec{m}_{(0)}\text{ .}$$

Knowing that the mean of the mRNA distribution is related to the vector of partial moments by: $\left\langle m\right\rangle=\vec{u}\text{ }\vec{m}_{(1)}$, we find that:

$$\left\langle m\right\rangle=\frac{\vec{r}\text{ }\vec{m}_{(0)}}{\gamma}\text{ .}$$

Note that, by definition,

$$\vec{m}_{(0)}=\sum_{m=0}^{\infty} m^{0}\vec{p}(m)=\left(\begin{matrix} \sum_{m=0}^{\infty} m^{0}p(1,m) \\ \sum_{m=0}^{\infty} m^{0}p(2,m) \end{matrix}\right)=\left(\begin{matrix} p(1) \\ p(2) \end{matrix}\right)\text{ .}$$

In other words, the first element of vector $\vec{m}_{(0)}$ is the steady state probability to find the promoter in state 1, and the second element is the steady state probability to find the promoter in state 2. This vector is straightforward to obtain by summing equation (5) over all *m*, and it is the solution of , normalized so that $p(1)+p(2)=1$.

In order to find the second moment, we just multiply equation (5) by $m^{2}$and sum over all *m* from 0 to $\infty$. As a result of this manipulation, we find:

$$\begin{matrix} \sum_{m=0}^{\infty} m^{2}\left(\left[\hat{K}-\hat{R}-m\gamma\hat{I}\right]\vec{p}(m)+\hat{R}\text{ }\vec{p}(m-1)+(m+1)\gamma\hat{I}\text{ }\vec{p}(m+1)\right)=\sum_{m=0}^{\infty} m^{2}\text{ }\hat{K}\text{ }\vec{p}(m)-\sum_{m=0}^{\infty} m^{3}\text{ }\gamma\hat{I}\text{ }\vec{p}(m) \\ -\sum_{m=0}^{\infty} m^{2}\text{ }\hat{R}\text{ }\vec{p}(m)+\sum_{m=0}^{\infty} m^{2}\text{ }\hat{R}\text{ }\vec{p}(m-1)+\sum_{m=0}^{\infty} m^{2}\text{ }(m+1)\text{ }\gamma\hat{I}\text{ }\vec{p}(m+1)= \\ \hat{K}\text{ }\vec{m}_{(2)}-\gamma\hat{I}\text{ }\vec{m}_{(3)}-\hat{R}\text{ }\vec{m}_{(2)}+\sum_{m=0}^{\infty} m^{2}\text{ }\hat{R}\text{ }\vec{p}(m-1)+\sum_{m=0}^{\infty} m^{2}\text{ }(m+1)\text{ }\gamma\hat{I}\text{ }\vec{p}(m+1)\text{ .} \end{matrix}$$

The last two terms of the right hand side of equation (18) can be simplified by writing the two sums in terms of the vectors of partial moments. In order to do that, we must make the same changes of variables that we invoked above when dealing with the mean. First, the change of variables $m\to m+1$ allows us to rewrite the first sum as:

$$\sum_{m=0}^{\infty} m^{2}\text{ }\vec{p}(m-1)=\sum_{m=0}^{\infty} (m+1)^{2}\text{ }\vec{p}(m)=\vec{m}_{(2)}+2\vec{m}_{(1)}+\vec{m}_{(0)}\text{ .}$$

Finally, the change of variables , allows us to re-write the last sum as:

$$\sum_{m=0}^{\infty} m^{2}(m+1)\text{ }\vec{p}(m+1)=\sum_{m=0}^{\infty} m(m-1)^{2}\text{ }\vec{p}(m)=\vec{m}_{(3)}-2\vec{m}_{(2)}+\vec{m}_{(1)}\text{ .}$$

Entering these last two sums in equation (18), we find:

$$\begin{matrix} \hat{K}\text{ }\vec{m}_{(2)}-\gamma\hat{I}\text{ }\vec{m}_{(3)}-\hat{R}\text{ }\vec{m}_{(2)}+\hat{R}\left(\vec{m}_{(2)}+2\vec{m}_{(1)}+\vec{m}_{(0)}\right)+\gamma\hat{I}\left(\vec{m}_{(3)}-2\vec{m}_{(2)}+\vec{m}_{(1)}\right)= \\ \hat{K}\text{ }\vec{m}_{(2)}+\hat{R}\left(2\vec{m}_{(1)}+\vec{m}_{(0)}\right)+\gamma\hat{I}\left(-2\vec{m}_{(2)}+\vec{m}_{(1)}\right)=0\text{ .} \end{matrix}$$

As we did before, we can transform this equation into an equation for the moments of the mRNA distribution by multiplying both sides of this equation on the left by the vector . Performing these operations, we find:

$$\begin{matrix} \vec{u}\text{ }\hat{K}\text{ }\vec{m}_{(2)}+\vec{u}\text{ }\hat{R}\left(2\vec{m}_{(1)}+\vec{m}_{(0)}\right)+\vec{u}\text{ }\gamma\hat{I}\left(-2\vec{m}_{(2)}+\vec{m}_{(1)}\right)=\vec{r}\left(2\vec{m}_{(1)}+\vec{m}_{(0)}\right)+\gamma\vec{u}\text{ }\left(-2\vec{m}_{(2)}+\vec{m}_{(1)}\right)= \\ 2\text{ }\vec{r}\text{ }\vec{m}_{(1)}+\vec{r}\text{ }\vec{m}_{(0)}-2\text{ }\gamma\left\langle m^{2}\right\rangle+\gamma\left\langle m\right\rangle=0\text{ .} \end{matrix}$$

Therefore, the second moment of the mRNA distribution in steady state is given by:

$$\left\langle m^{2}\right\rangle=\frac{\vec{r}\text{ }\vec{m}_{(1)}}{\gamma}+\frac{\vec{r}\text{ }\vec{m}_{(0)}+\gamma\left\langle m\right\rangle}{2\gamma}\text{ .}$$

Using the fact that the first moment is given by:
$\left\langle m\right\rangle=\frac{\vec{r}\text{ }\vec{m}_{(0)}}{\gamma}\text{ .}$

We can further simplify the second moment as:

$$\left\langle m^{2}\right\rangle=\left\langle m\right\rangle+\frac{\vec{r}\text{ }\vec{m}_{(1)}}{\gamma}\text{ .}$$

Therefore, the normalized variance can be written as:

$$\eta^{2}=\frac{\left\langle m^{2}\right\rangle-\left\langle m\right\rangle^{2}}{\left\langle m\right\rangle^{2}}=\frac{1}{\left\langle m\right\rangle}+\frac{1}{\left\langle m\right\rangle^{2}}\left(\frac{\vec{r}\text{ }\vec{m}_{(1)}}{\gamma}-\left\langle m\right\rangle^{2}\right)\text{ .}$$

**The moments of the protein probability distribution**

We can use the same method to compute the normalized variance of the protein distribution. We will start from a promoter that is constitutively active, and then extend our analysis to a promoter that switches between two or more active and inactive states. We assume that each transcription event leads to the production of multiple proteins (a “burst”). The number of proteins produced per mRNA (which we denote as $\beta$) obeys a geometric distribution [3,4,5] with an average burst size $b$. Therefore, the probability for $\beta$ is given by: $h\left(\beta\right)=\frac{b^{\beta}}{\left(1+b\right)^{\beta+1}}$. We assume that proteins are also degraded with a constant rate per molecule of $\delta$. In order to write down the master equation for this process, we have to consider all the possible ways in which the cell can enter or leave a state with *n* proteins during a small increment of time dt. If we assume that mRNA lifetime is much shorter than protein lifetime (an approximation that is realistic in many experimental systems –see refs [4,5,6]), then all of the proteins may be assumed to be made simultaneously. Therefore, we need to consider the possibility that the cell will jump from a state with *n* proteins to a state with $n+\beta$, for all possible values of $\beta$. The probability that the cell will leave a state with *n* proteins, by making a transition to a state with $n+\beta$ proteins is equal to the product of the probability that the cell is in a state with *n* proteins$\left(p(n)\right)$, the probability that the cell will make a transcript during *dt* $\left(rdt\right)$, and the probability that the transcript makes $\beta$proteins before it is degraded $\left(h\left(\beta\right)\right)$. Thus, the total probability per unit time to abandon a state with *n* proteins is given by $r\text{ }h\left(\beta\right)p(n)$. Since $\beta$ can in principle take any integer value, the total probability to abandon the state with *n* proteins by the occurrence of a protein burst is given by the sum of $r\text{ }h\left(\beta\right)p(n)$over all possible values of $\beta$. This term will be given by: $\sum_{\beta=1}^{\infty} r\text{ }h\left(\beta\right)p(n)=r\text{ }p(n)\sum_{\beta=1}^{\infty} h\left(\beta\right)$. Also, we need to consider that the cell may enter a state with *n* proteins from any state with less than *n* proteins. The probability per unit time that the cell enters a state with n proteins, from a state with $n-\beta$proteins is given by:$rh\left(\beta\right)p(n-\beta)$. Therefore, following the same logic as we did before, the net probability per unit time that the cell enters a state with *n* proteins is $\sum_{\beta=1}^{n} r\text{ }h\left(\beta\right)p(n-\beta)$. With these considerations, the master equation for a constitutive promoter is given by:

$$\frac{d}{dt}p(n)=-\sum_{\beta=1}^{\infty} rh\left(\beta\right)p(n)+\sum_{\beta=1}^{n} rh\left(\beta\right)p(n-\beta)-\delta np(n)+\delta(n+1)p(n+1)\text{ .}$$

As discussed above, the first sum can be further simplified to:

$\sum_{\beta=1}^{\infty} rh\left(\beta\right)p(n)=r\text{ }p(n)\sum_{\beta=1}^{\infty} h\left(\beta\right)=r\text{ }p(n)\sum_{\beta=1}^{\infty} \frac{b^{\beta}}{\left(1+b\right)^{\beta+1}}=r\left(\frac{b}{1+b}\right)p(n)$.

As a result, the master equation takes the form:

$\frac{d}{dt}p(n)=-r\left(\frac{b}{1+b}\right)p(n)+\sum_{\beta=1}^{n} rh\left(\beta\right)p(n-\beta)-\delta np(n)+\delta(n+1)p(n+1)$.

In steady state, the right hand side of equation (29) is equal to 0, and we have:

$0=-r\left(\frac{b}{1+b}\right)p(n)+\sum_{\beta=1}^{n} rh\left(\beta\right)p(n-\beta)-\delta np(n)+\delta(n+1)p(n+1)$.

The first two moments of the steady state protein distribution $p(n)$can be obtained, in exactly the same way we used to find out the moments of the mRNA distribution in the previous section: by multiplying both sides of equation (30) by $n$and $n^{2}$ respectively, and then summing over all $n$. Before we do that, it is useful to evaluate the sums $\sum_{n=0}^{\infty} n^{2}\sum_{\beta=1}^{n} rh\left(\beta\right)p(n-\beta)$ and $\sum_{n=0}^{\infty} n\sum_{\beta=1}^{n} rh\left(\beta\right)p(n-\beta)$. We can find the general term of the first sum by expanding the series:

$$\begin{matrix} \sum_{n=0}^{\infty} n^{2}\sum_{\beta=1}^{n} h\left(\beta\right)p(n-\beta)=1^{2}\left(h(1)p(0)\right)+2^{2}\left(h(1)p(1)+h(2)p(0)\right)+3^{2}\left(h(1)p(2)+h(2)p(1)+h(3)p(0)\right)+...= \\ \left(1^{2}h(1)+2^{2}h(2)+3^{2}h(3)...\right)p(0)+\left(2^{2}h(1)+3^{2}h(2)+4^{2}h(3)...\right)p(1)+\left(3^{2}h(1)+4^{2}h(2)+5^{2}h(3)...\right)p(2)+...= \\ \sum_{n=0}^{\infty} p(n)\left(\sum_{\beta=1}^{\infty} h\left(\beta\right)\left(n+\beta\right)^{2}\right)=\sum_{n=0}^{\infty} \left(b+2b^{2}+2bn+\frac{b}{1+b}n^{2}\right)p(n)\text{ .} \end{matrix}$$

We can do the same for the second sum, and we find:

$$\begin{matrix} \sum_{n=0}^{\infty} n\sum_{\beta=1}^{n} h\left(\beta\right)p(n-\beta)=1\left(h(1)p(0)\right)+2\left(h(1)p(1)+h(2)p(0)\right)+3\left(h(1)p(2)+h(2)p(1)+h(3)p(0)\right)+...= \\ \left(h(1)+2h(2)+3h(3)...\right)p(0)+\left(2h(1)+3h(2)+4h(3)...\right)p(1)+\left(3h(1)+4h(2)+5h(3)...\right)p(2)+...= \\ \sum_{n=0}^{\infty} p(n)\left(\sum_{\beta=1}^{\infty} h\left(\beta\right)\left(n+\beta\right)\right)=\sum_{n=0}^{\infty} \left(b+\frac{b}{1+b}n\right)p(n)\text{ .} \end{matrix}$$

Likewise, it will be necessary to recall from the first section of this supplement, that the sum $\sum_{n=0}^{\infty} n(n+1)p(n+1)$ can be computed by using the change of variables: $n+1\to n$, and we find: $\sum_{n=0}^{\infty} n(n+1)p(n+1)=\sum_{n=0}^{\infty} n(n-1)p(n)$ .

With these results in hand, we can finally solve the first two moments of the protein distribution $p(n)$. As explained above, we can find the first moment by multiplying both sides of equation (30) by $n$and then summing over all *n*. In order to find the second moment, we multiply both sides of equation (30) by $n^{2}$and then sum over all *n*. For the first moment, we find:

$$\begin{matrix} 0=-r\left(\frac{b}{1+b}\right)\sum_{n=0}^{\infty} np(n)+r\sum_{n=0}^{\infty} n\sum_{\beta=1}^{n} h\left(\beta\right)p(n-\beta)-\delta\sum_{n=0}^{\infty} n^{2}p(n)+\delta\sum_{n=0}^{\infty} n(n+1)p(n+1)= \\ \text{ }=-r\left(\frac{b}{1+b}\right)\left\langle n\right\rangle+r\left(b+\frac{b}{1+b}\left\langle n\right\rangle\right)-\delta\left\langle n^{2}\right\rangle+\delta\left\langle n^{2}\right\rangle-\delta\left\langle n\right\rangle=rb-\delta\left\langle n\right\rangle. \end{matrix}$$

Solving this equation, we find that the mean protein per cell is equal to:

$\left\langle n\right\rangle=\frac{rb}{\delta}$.

For the second moment, we find:

$$\begin{matrix} 0=-r\left(\frac{b}{1+b}\right)\sum_{n=0}^{\infty} n^{2}p(n)+r\sum_{n=0}^{\infty} n^{2}\sum_{\beta=1}^{n} h\left(\beta\right)p(n-\beta)-\delta\sum_{n=0}^{\infty} n^{3}p(n)+\delta\sum_{n=0}^{\infty} n^{2}(n+1)p(n+1)= \\ \text{ }=-r\left(\frac{b}{1+b}\right)\left\langle n\right\rangle+r\left(b+2b^{2}+2b\left\langle n\right\rangle+\frac{b}{1+b}\left\langle n^{2}\right\rangle\right)-\delta\left\langle n^{3}\right\rangle+\delta\left\langle n^{3}\right\rangle-2\delta\left\langle n^{2}\right\rangle+\delta\left\langle n\right\rangle= \\ \text{ }=r\left(b+2b^{2}+2b\left\langle n\right\rangle\right)-2\delta\left\langle n^{2}\right\rangle+\delta\left\langle n\right\rangle. \end{matrix}$$

Solving this last equation, we find that the second moment of the protein distribution is equal to:

$\left\langle n^{2}\right\rangle=\frac{r}{2\delta}b+\frac{r}{2\delta}2b^{2}+\frac{r}{2\delta}2b\left\langle n\right\rangle+\frac{\left\langle n\right\rangle}{2}=\left(1+b\right)\left\langle n\right\rangle+\left\langle n\right\rangle^{2}$.

Therefore, the normalized variance of the protein distribution for a constitutive promoter takes the form:

$$\frac{Var(n)}{\left\langle n\right\rangle^{2}}=\frac{\left\langle n^{2}\right\rangle-\left\langle n\right\rangle^{2}}{\left\langle n\right\rangle^{2}}=\frac{\left(1+b\right)\left\langle n\right\rangle+\left\langle n\right\rangle^{2}-\left\langle n\right\rangle^{2}}{\left\langle n\right\rangle^{2}}=\frac{\left(1+b\right)}{\left\langle n\right\rangle}\text{ .}$$

If now we consider that the promoter can exist in two states, characterized by having different rates of transcription, then the cell’s state is characterized not only by the number of proteins present, but also by the state of the promoter. Therefore, the master equation must consider two variables: one characterizing the state of the promoter (*s*), and one representing the number of proteins per cell (*n*). By analogy with the mRNA master equation, and the master equation for the protein distribution of a constitutive promoter, the two-state master equation for the protein distribution can be written as:

$$\begin{matrix} \frac{d}{dt}p(1,n)=-k_{A}^{on}p(1,n)+k_{A}^{off}p(2,n)-\sum_{\beta=1}^{\infty} r_{1}h\left(\beta\right)p(1,n)+\sum_{\beta=1}^{n} r_{1}h\left(\beta\right)p(1,n-\beta)-\delta np(1,n)+\delta(n+1)p(1,n+1), \\ \frac{d}{dt}p(2,n)=k_{A}^{on}p(1,n)-k_{A}^{off}p(2,n)-\sum_{\beta=1}^{\infty} r_{2}h\left(\beta\right)p(2,n)+\sum_{\beta=1}^{n} r_{2}h\left(\beta\right)p(2,n-\beta)-\delta np(2,n)+\delta(n+1)p(2,n+1)\text{ .} \end{matrix}$$

Just as we did in order to compute the moments of the mRNA distribution, we can define the vector $\vec{p}(n)=\left(p(1,n),p(2,n)\right)$. By doing so, we will be able to re-write the master equation (39) as a matrix equation, that will be applicable to any promoter with any number of states. This matrix equation can be written in terms of exactly the same matrices we used for the mRNA probability distribution. We find:

$$\frac{d}{dt}\vec{p}(n)=\left[\hat{K}-\frac{b}{1+b}\hat{R}-n\delta\text{ }\hat{I}\right]\vec{p}(n)+\hat{R}\sum_{\beta=1}^{n} h(\beta)\vec{p}(n-\beta)+(n+1)\delta\text{ }\hat{I}\text{ }\vec{p}(n+1)\text{ .}$$

In steady state, the left side of equation (40) is equal to 0, and the master equation has the form:

$$0=\left[\hat{K}-\frac{b}{1+b}\hat{R}-n\delta\text{ }\hat{I}\right]\vec{p}(n)+\hat{R}\sum_{\beta=1}^{n} h(\beta)\vec{p}(n-\beta)+(n+1)\delta\text{ }\hat{I}\text{ }\vec{p}(n+1)\text{ .}$$

Just as we did in order to calculate the moments of the mRNA distribution, it will be convenient to define the vectors of partial moments:

$$\begin{matrix} \vec{n}_{(0)}=\sum_{n=0}^{\infty} n^{0}\vec{p}(n)=\left(\begin{matrix} \sum_{n=0}^{\infty} n^{0}p(1,n) \\ \sum_{n=0}^{\infty} n^{0}p(2,n) \end{matrix}\right)=\left(\begin{matrix} p(1) \\ p(2) \end{matrix}\right), \\ \vec{n}_{(1)}=\sum_{n=0}^{\infty} n\text{ }\vec{p}(n)=\left(\begin{matrix} \sum_{n=0}^{\infty} n\text{ }p(1,n) \\ \sum_{n=0}^{\infty} n\text{ }p(2,n) \end{matrix}\right), \\ \vec{n}_{(2)}=\sum_{n=0}^{\infty} n^{2}\text{ }\vec{p}(n)=\left(\begin{matrix} \sum_{n=0}^{\infty} n^{2}\text{ }p(1,n) \\ \sum_{n=0}^{\infty} n^{2}\text{ }p(2,n) \end{matrix}\right)\text{ .} \end{matrix}$$

It is straightforward to see that the vector$\vec{n}_{(0)}$ is exactly identical to the vector $\vec{m}_{(0)}$. The next two vectors $\vec{n}_{(1)}$and $\vec{n}_{(2)}$can be obtained by multiplying equation (41) by $n$and $n^{2}$respectively, and then summing over all n. We end up with the following two equations:

$$\begin{matrix} 0=\sum_{n=0}^{\infty} n\left[\hat{K}-\frac{b}{1+b}\hat{R}-n\delta\text{ }\hat{I}\right]\vec{p}(n)+\hat{R}\sum_{n=0}^{\infty} n\sum_{\beta=1}^{n} h(\beta)\vec{p}(n-\beta)+\sum_{n=0}^{\infty} n(n+1)\delta\text{ }\hat{I}\cdot\vec{p}(n+1)= \\ \text{ }=\hat{K}\text{ }\vec{n}_{(1)}-\frac{b}{1+b}\hat{R}\text{ }\vec{n}_{(1)}-\delta\text{ }\hat{I}\text{ }\vec{n}_{(2)}+\delta\text{ }\hat{I}\left(\vec{n}_{(2)}-\vec{n}_{(1)}\right)+\hat{R}\left(\frac{b}{1+b}\vec{n}_{(1)}+b\vec{n}_{(0)}\right) \\ \text{ }=\left(\hat{K}-\delta\text{ }\hat{I}\right)\text{ }\vec{n}_{(1)}+b\text{ }\hat{R}\text{ }\vec{n}_{(0)}, \end{matrix}$$

and

$$\begin{matrix} 0=\sum_{n=0}^{\infty} n^{2}\left[\hat{K}-\frac{b}{1+b}\hat{R}-n\delta\text{ }\hat{I}\right]\vec{p}(n)+\hat{R}\sum_{n=0}^{\infty} n^{2}\sum_{\beta=1}^{n} h(\beta)\vec{p}(n-\beta)+\sum_{n=0}^{\infty} n^{2}(n+1)\delta\text{ }\hat{I}\cdot\vec{p}(n+1)= \\ \text{ }=\hat{K}\text{ }\vec{n}_{(2)}-\frac{b}{1+b}\hat{R}\text{ }\vec{n}_{(2)}-\delta\text{ }\hat{I}\text{ }\vec{n}_{(3)}+\delta\text{ }\hat{I}\left(\vec{n}_{(3)}-2\vec{n}_{(2)}+\vec{n}_{(1)}\right)+\hat{R}\left(\frac{b}{1+b}\vec{n}_{(2)}+2b\vec{n}_{(1)}+b(1+2b)\vec{n}_{(0)}\right)= \\ \text{ }=\hat{K}\text{ }\vec{n}_{(2)}+\delta\hat{I}\left(-2\vec{n}_{(2)}+\vec{n}_{(1)}\right)+\hat{R}\left(2b\vec{n}_{(1)}+b(1+2b)\vec{n}_{(0)}\right)= \\ \text{ }=\left(\hat{K}\text{ }-2\delta\hat{I}\right)\vec{n}_{(2)}+\left(\delta\hat{I}+2b\hat{R}\right)\vec{n}_{(1)}+b(1+2b)\hat{R}\text{ }\vec{n}_{(0)}\text{ .} \end{matrix}$$

Now by multiplying the vector $\vec{u}=\left(1,1\right)$ on the left of equations (43) and (44), we find

$0=-\delta\left\langle n\right\rangle+b\text{ }\vec{r}\text{ }\vec{n}_{(0)}$,

and:

$$0=-2\delta\left\langle n^{2}\right\rangle+\delta\left\langle n\right\rangle+2b\text{ }\vec{r}\text{ }\vec{n}_{(1)}+b(1+2b)\vec{r}\text{ }\vec{n}_{(0)}\text{ .}$$

Thus, we find analytical equations for the first two moments of the protein distribution:

$$\left\langle n\right\rangle=\frac{b\text{ }\vec{r}\text{ }\vec{n}_{(0)}}{\delta},$$

$$\left\langle n^{2}\right\rangle=\left(1+b\right)\left\langle n\right\rangle+\frac{b\text{ }\vec{r}\text{ }\vec{n}_{(1)}}{\delta}.$$

Where $\vec{n}_{(1)}$ is the solution of equation (43):

$$0=\left(\hat{K}-\delta\text{ }\hat{I}\right)\text{ }\vec{n}_{(1)}+b\text{ }\hat{R}\text{ }\vec{n}_{(0)},$$

Armed with these equations, we can finally compute the stationary variance of the protein distribution:

$$\frac{Var(n)}{\left\langle n\right\rangle^{2}}=\frac{\left(1+b\right)\left\langle n\right\rangle+\frac{b\text{ }\vec{r}\cdot\vec{n}_{(1)}}{\delta}-\left\langle n\right\rangle^{2}}{\left\langle n\right\rangle^{2}}=\frac{\left(1+b\right)}{\left\langle n\right\rangle}+\frac{1}{\left\langle n\right\rangle^{2}}\left(\frac{b\text{ }\vec{r}\text{ }\vec{n}_{(1)}}{\delta}-\left\langle n\right\rangle^{2}\right)\text{ .}$$

**Exploration of the space of parameter values**

In order to test how some of the key qualitative and quantitative conclusions discussed in the main text depend on choice of rate constants that characterize the different architectures, we computed the Fano factor for a large set of parameter values drawn randomly from the space of possible values. The results of these calculations are shown in figures S2, S3, and S4.

**REFERENCES**

1. Sanchez A, Kondev J (2008) Transcriptional control of noise in gene expression. Proc Natl Acad Sci: 105,5081-5086.

2. Kepler TB, Elston TC (2001) Stochasticity in transcriptional regulation: origins, consequences, and mathematical representations. Biophys J: 81,3116-3136.

3. Berg O (1978) A model for statistical fluctuations of protein numbers in a microbial-population. J Theor Biol: 71:587-603.

4. Cai L, Friedman N, Xie XS (2006) Stochastic protein expression in individual cells at the single molecule level. Nature: 440, 358-362.

5. Yu J, Xiao, J., Ren, X., Lao, K., S. Xie (2006) Probing gene expression in live cells one protein at a time. Science: (311)1600-1603.

6. Kennell D, Riezman, H. (1977) Transcription and translation initiation frequencies of the Escherichia coli lac operon. J Mol Biol 114: 1-21.

**SUPPLEMENTARY FIGURES**

**
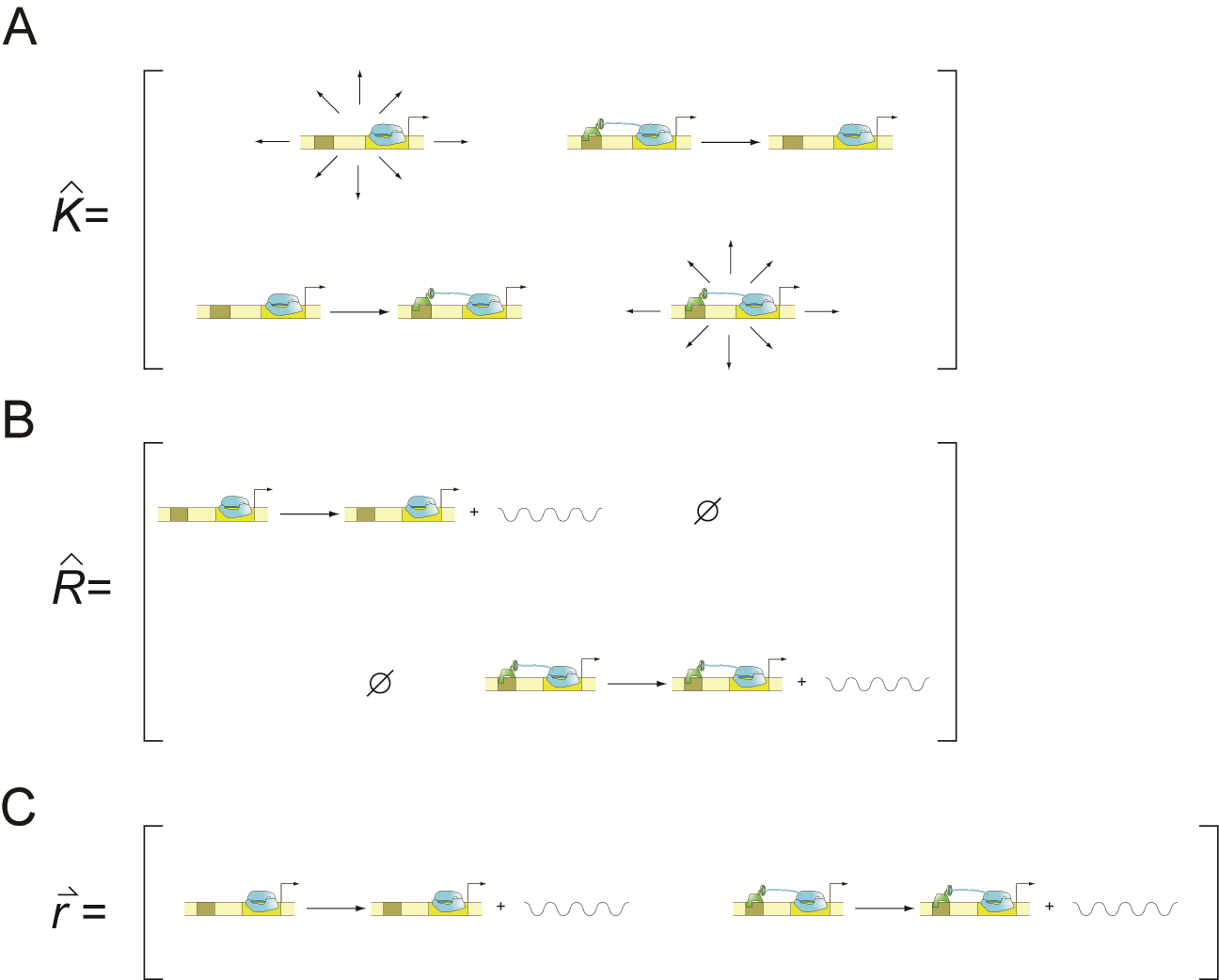
**

**Figure 1. Cartoon depiction of the construction of kinetic rate matrices and vectors.** (A) Cartoon representation of the kinetic rate matrix $\hat{K}$. The diagonal elements represent the net rate at which the promoter abandons each state. For instance, element $\left\{\hat{K}\right\}_{11}$ is the rate at which the promoter abandons state 1 due to stochastic association of the activator with the promoter: $\left\{\hat{K}\right\}_{11}=-k_{A}^{on}$, and element $\left\{\hat{K}\right\}_{22}=-k_{A}^{off}$ is the rate of dissociation of the activator from the promoter, abandoning state 2. The non-diagonal element $\left\{\hat{K}\right\}_{21}=k_{A}^{on}$ is the rate at which the promoter makes a transition from state 1 to state 2 (by dissociation association of one activator to the promoter), and the non-diagonal element $\left\{\hat{K}\right\}_{12}=k_{A}^{off}$ is the rate at which the promoter makes a transition from state 2 to state 1 (by dissociation of the activator). (B) The transcription rate matrix $\hat{R}$contains, in its diagonal elements, the net rate of transcription at each promoter state. Element $\left\{\hat{R}\right\}_{11}=r_{1}$ is the rate of transcription in promoter state 1 and$\left\{\hat{R}\right\}_{22}=r_{2}$ is the rate of transcription in promoter state 2. (C) The vector $\vec{r}=\left(r_{1},r_{2}\right)$ contains the rates of transcription at states 1 and 2, and is identical to the diagonal of matrix $\hat{R}$.

**
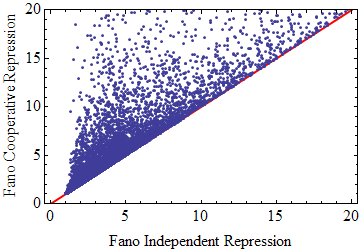
**

**Figure 2. Effect of parameter choice on Fano factor for independent and cooperative repression architectures.** We sample the parameter space by randomly selecting 10,000 different values for the mean mRNA $\left\langle m\right\rangle$ (within 0.005 and 100), $k_{R}^{off}$ (from $k_{R}^{off}/\gamma$=0.01 to $k_{R}^{off}/\gamma$=100), $\left\langle m\right\rangle_{\max}$ (from 5 to 100), and $\Omega$(from 0.001 to 1). The Fano factor is calculated for both independent and cooperative repression architectures, when the mean is the same for both. In the X axis we plot the Fano Factor for independent repression. In the Y axis we plot the Fano factor for cooperative repression. As is the case throughout the paper, we assume that we vary the mean by titrating the amount of repressor inside the cell. Each point in the figure corresponds to two architectures with the same mean. We find that cooperative binding always results in larger cell-to-cell variability than non-cooperative binding. The red solid line marks the region where the Fano factor is the same for both architectures.

**
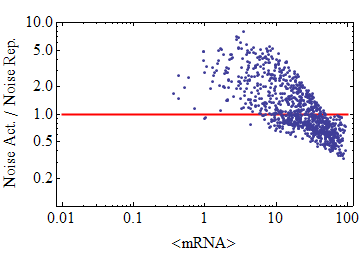
**

**Figure 3. Simple activation tends to be noisier than simple repression at low expression levels.** We follow the same procedure as in figure S1, and sample the parameter space by randomly selecting 1,000 different values for the mean mRNA $\left\langle m\right\rangle$ (within 0.01 and 100), $k_{R}^{off}$and $k_{A}^{off}$ (from 0.01 γ to 100 γ), $\left\langle m\right\rangle_{\max}$, the enhancement factor $f$ (from 10 to 100). For each one of these 10,000 sets of parameters, we compute the Fano factor for the simple activation and the simple repression architectures. We plot the ratio between the Fano factor for simple activation and repression as a function of the mean. We find that at low mRNA levels $\left(\left\langle m\right\rangle<10\right)$, the simple activation architecture is noisier than the simple repression architecture in over 99% of the sets of rates tested here. In contrast, at high mRNA levels, it is the other way around. In order for the comparison between both architectures to be meaningful, we have assumed that the repressor and the activator have the same affinity for their operators (even if we vary this affinity over 4 orders of magnitude). The red solid line marks the region where the Fano factor is the same for both architectures (and thus the ratio between the two is 1)


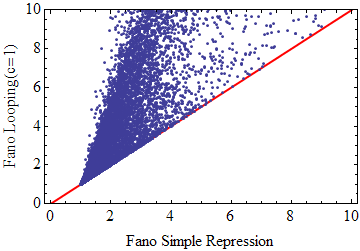

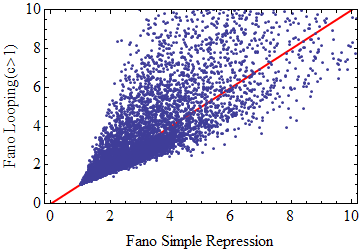


A

B

**Figure 4. Effect of parameter choice on Fano factor for the repression by DNA looping architecture** We sample the parameter space by randomly selecting 10,000 different values for the mean mRNA $\left\langle m\right\rangle$ (within 0.005 and 100), $k_{R}^{off}$ (from $k_{R}^{off}/\gamma$=0.01 to $k_{R}^{off}/\gamma$=100), $\left\langle m\right\rangle_{\max}$ (from 5 to 100), $k_{loop}$(from 0.01 γ to 100 γ), and the parameter characterizing the rate of dissociation in the presence of the auxiliary operator, relative to that in its absence (*c*). We first assume that *c* = 1 for all parameter sets (A), and then we randomly sample it within 1 and 10 (B). In the X axis we plot the Fano Factor for simple repression. In the Y axis we plot the Fano factor for repression by DNA looping. As is the case throughout the paper, we assume that we vary the mean by titrating the amount of repressor inside the cell. Each point in the figure corresponds to two architectures with the same mean. We find that whether DNA looping enhances or diminishes noise depends on the value of *c*. If *c* = 1, meaning that DNA looping does not affect the rate of dissociation of the repressor from the operator, the Fano factor for the DNA looping architecture is larger than the Fano factor for the simple repression architecture. On the other hand, if $c>1$, DNA looping may decrease noise (as observed for ~40% of the parameters chosen). The red solid line marks the region where the Fano factor is the same for both architectures.

**
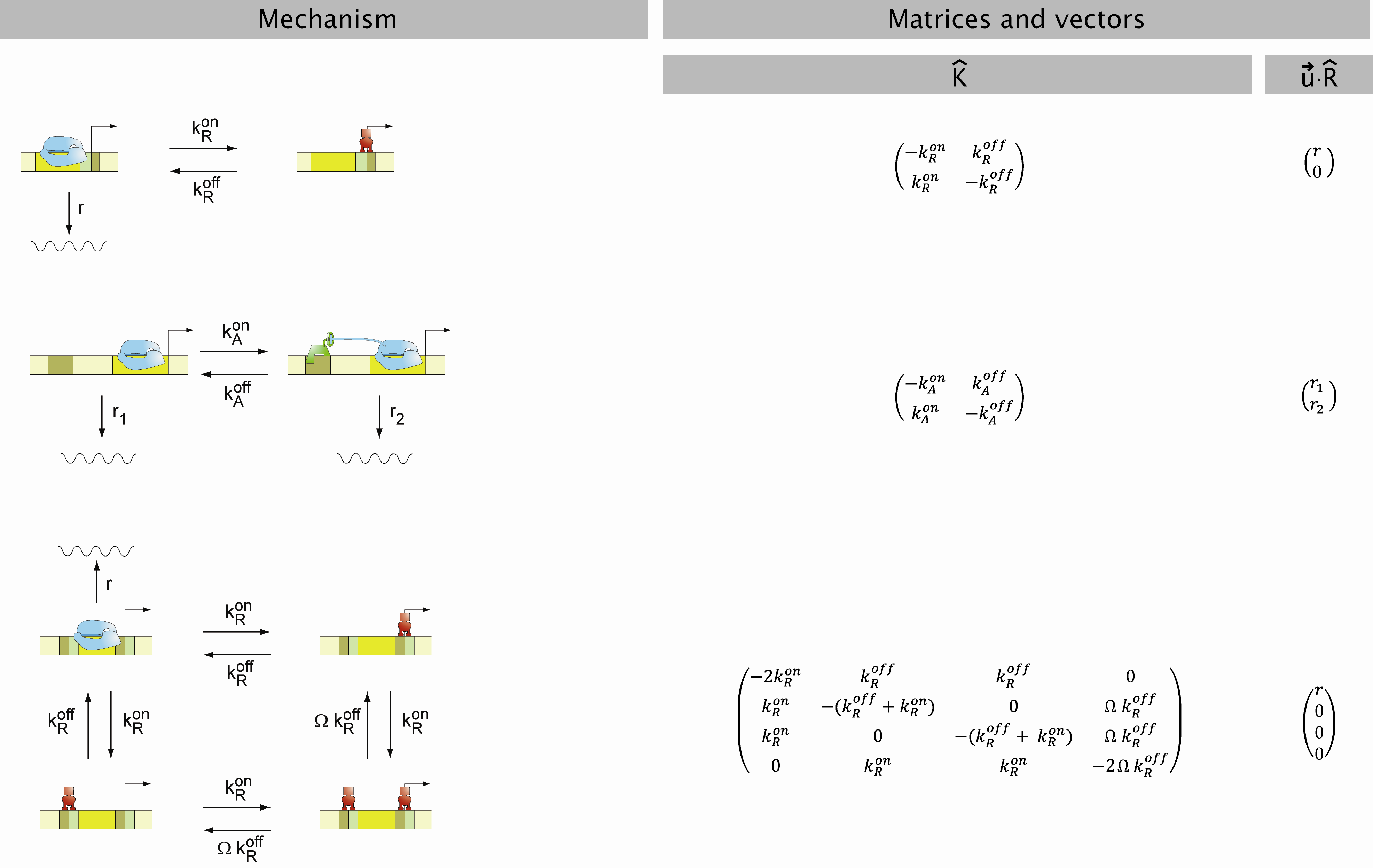
**

**
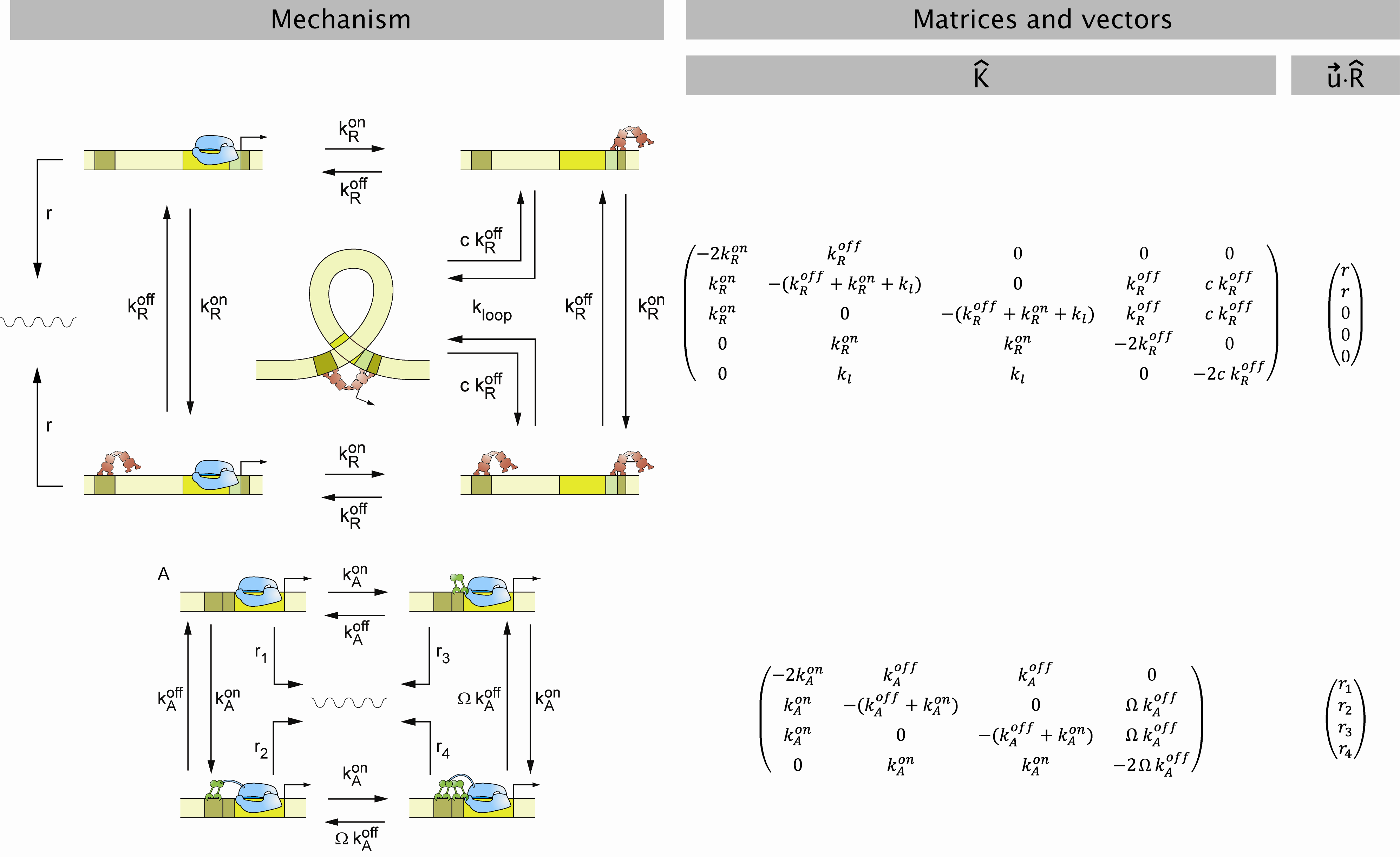
**

**Table S1: Kinetic rate matrices for all mechanisms in the text.** In the first column, we represent the kinetic mechanisms of gene regulation for all of the architectures considered in the text. In the second and third columns, we show the corresponding promoter kinetic transition rate matrices and the vector for all of the mechanisms.


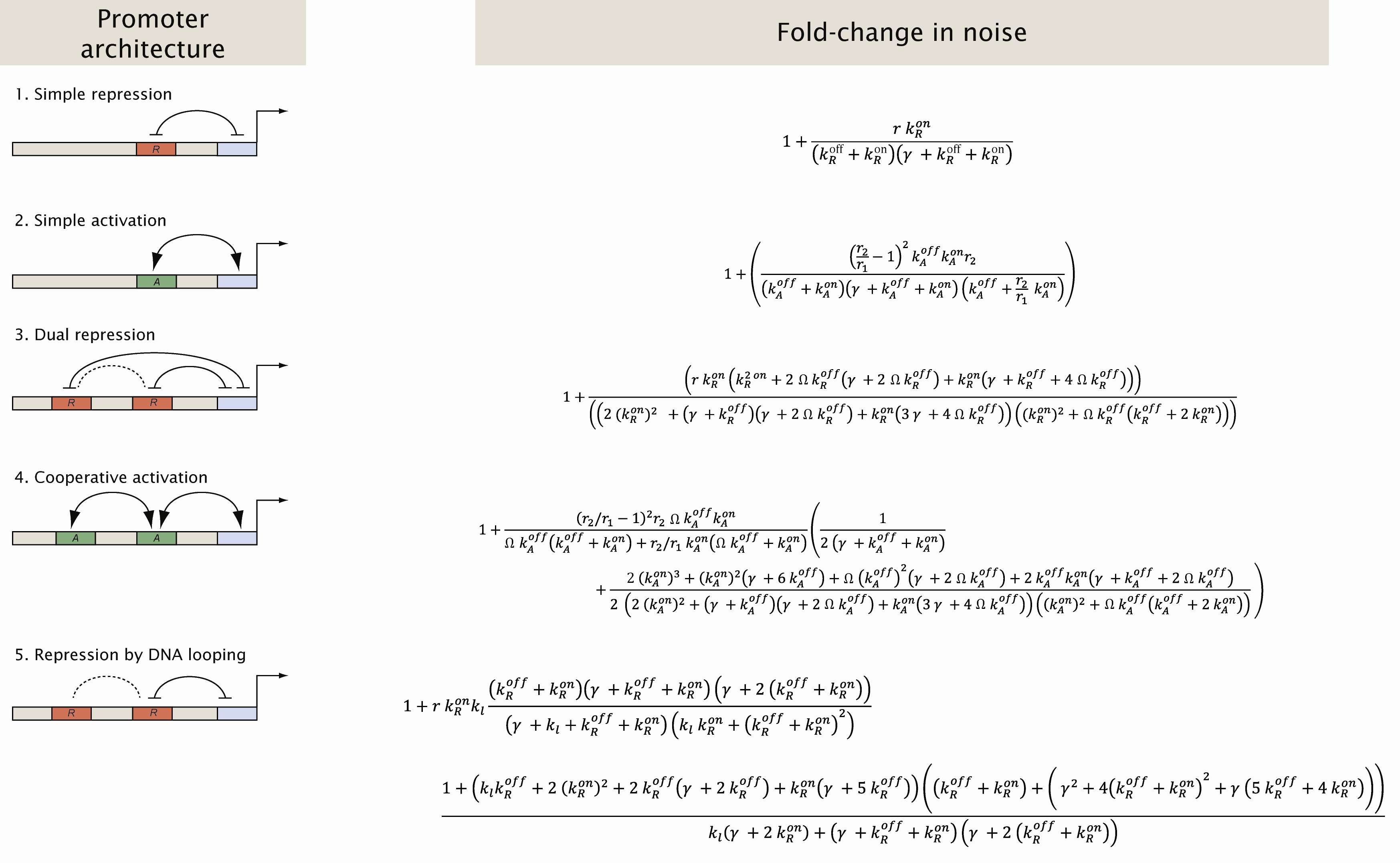


**Table S2: Fold-change in noise for different promoter architectures.** The fold-change in promoter noise is shown as a function of the different kinetic parameters corresponding to each promoter architecture considered throughout the text. Refer to Table I for the definition and value of each rate.
